# Parvovirus B19 Rebound

**DOI:** 10.1002/jmv.70380

**Published:** 2025-05-03

**Authors:** Stefania Ranno, Cristina Russo, Luna Colagrossi, Valeria Fox, Velia Chiara Di Maio, Giulia Linardos, Leonarda Gentile, Rosaria Marotta, Sebastian Cristaldi, Andrea Campana, Mara Pisani, Leonardo Caforio, Alberto Villani, Carlo Federico Perno, Luana Coltella

**Affiliations:** ^1^ Microbiology and Diagnostic Immunology Unit, Bambino Gesù Children's Hospital, IRCCS Rome Italy; ^2^ Pediatric Emergency Unit, Bambino Gesù Children's Hospital, IRCCS Rome Italy; ^3^ Pediatrics Unit, Bambino Gesù Children's Hospital, IRCCS Rome Italy; ^4^ Fetal Medicine and Surgery Unit, Bambino Gesù Children's Hospital, IRCCS Rome Italy

**Keywords:** B19 virus, epidemiology, immunoglobulin, infection, parvovirus

## Abstract

Human parvovirus B19 (B19V) is responsible for a wide clinical spectrum ranging from asymptomatic infection, through mild disease, up to life‐threatening one. Outbreaks are registered every 3–4 years, and a recent international alert for a new outbreak has been released. The experience of B19 virus circulation in a 600‐bed tertiary care pediatric hospital in Rome from 2018 to 2024 is reported here. This retrospective study involved a total of 9695 blood samples (about 8500 patients), 11 amniotic fluids (11 pregnant women), and 10 827 sera (about 9500 patients), processed in the Virology Unit of Bambino Gesù Children's Hospital in Rome for B19V direct and indirect detection. In our population, the annual positivity rate for B19V DNA ranged from 0.8% in 2023 to 9.8% in 2018 and 32.8% in 2024; the same trend resulted from the analysis of the immunoglobulins M and G anti‐B19V. Focusing on the last year, 314 patients resulted positive for B19V DNA detection: 204/314 (65%) had a primary infection, 150/204 (73.5%) were hospitalized, and 17/150 (11.3%) needed Intensive Care Unit (ICU) for cardiovascular, central nervous, and gastrointestinal pathologies. Two patients died from myocarditis. Among patients with the most severe clinical picture, over half had no concurring disease, and one patient died. Four amniotic fluids were positive for pregnant women who came to our observation. B19V typing of a subset of samples revealed the presence of only subtype 1A and a low intragenotypic diversity between strains from severe and mild disease. In conclusion, in 2024, a significant increase in B19V circulation was observed with profound effects on clinical outcome and consequent hospitalisation, either in patients with comorbidities or those without. This widespread circulation of the virus also had an impact on infections in pregnancy, with the known severe consequences for unborn children.

**Abbreviation**
ECMO: extracorporeal membrane oxygenation

## 1 Background

Human parvovirus B19 (B19V) is a single‐stranded DNA virus of the family Parvoviridae, genus *Erythrovirus*, first identified in 1975. Three genotypes of B19 have been described (genotypes 1–3) [1]. All three genotypes have been found in symptomatic as well as asymptomatic individuals and have been reported from several countries across the world [2].

B19V is responsible for a wide clinical spectrum ranging from asymptomatic infection, through to a mild disease, up to a life‐threatening one, mainly related to age, immunocompetence, and pregnancy status [3]. In children, B19V infection runs benign, classically causing *erythema infectiosum* (EI or fifth disease), a biphasic fever and rash illness characterized by prodromal nonspecific symptoms as fever, chills, headache, malaise, and myalgia, associated with the viremic phase, followed by the typical erythema, consequence of the immune response [4]. In adults, the typical EI rash is much less common [5], the cutaneous manifestations are polymorphous, and arthralgia predominates, particularly in women. Symmetrical, painful, swollen joints, especially wrists, knees, and hands, can last for weeks to months [6]. In immunocompromised patients, B19V infection is more likely to develop severe forms with myocardial and central nervous system complications [4]. These same patients are more prone to B19V‐induced chronic anemia [7]. In patients with hemoglobinopathies or red blood cell disorders, B19V infection may cause transient aplastic crisis or severe anemia [8, 9]. In pregnant women, B19V infection may be responsible for spontaneous abortion, stillbirth, hydrops fetalis, and severe anemia [4, 10]. Moreover, several reports suggest that B19V may contribute to the inflammation and autoimmune mechanisms involved in the pathogenesis of some rheumatologic diseases [11].

B19V infection usually occurs in childhood and continues at lower rates throughout adulthood, such that 70%–85% of adults are antibody positive [4].

Specific M immunoglobulins (IgM) anti‐B19V appear 10–12 days after B19V primary infection and may be found in serum for 3–6 months; G immunoglobulins (IgG) anti‐B19V appear 2 weeks after exposure and presumably persist for life, with an increase in their level after re‐exposure [7].

Transmission occurs via the respiratory route, it is year‐round, but may peak late winter to early summer, without ethnic or geographical boundaries [12]. Outbreaks of B19V activity are registered every 3–4 years [4]. Recently, ECDC released a document highlighting the increased circulation of Parvovirus across the whole Europe (*Risks posed by reported increased circulation of human parvovirus B19 in the EU/EEA, ECDC 2024*).

Here is the Bambino Gesù Children's Hospital experience about B19V circulation during the last years, from 2018 to 2024, focusing on the 2024 rebound. The study aims to present a comprehensive epidemiological picture of B19V circulation, detailing the infection trend, thanks to the different contributions of molecular and serological laboratory data. Moreover, the impact of B19V infection on a predominantly pediatric population is described, focusing on the most severe clinical outcomes.

## 2 Methods

This retrospective study involved samples belonging to inpatients and outpatients afferent to Bambino Gesù Children's Hospital in Rome and processed in the Virology Unit for B19V direct and indirect detection. No patient selection was performed, all samples with a request for B19 detection were included in this analysis to avoid any bias, but to have a clearer picture of B19V epidemiology, for molecular analysis, only blood samples and amniotic fluids were considered.

In particular, from January 1, 2018, to December 31, 2024, a total of 9695 blood samples, referring to about 8500 patients (pts), were analyzed for the search of Parvovirus B19 DNA, and 10 827 serum samples, referring to about 9500 pts, were processed for antibody against Parvovirus B19. The study also included 11 amniotic fluid samples, referring to 11 women with high‐risk pregnancy, followed by the Fetal and Perinatal Medicine and Surgery Center of our hospital between January 1, 2023 and December 31, 2024, investigated for B19V DNA.

Blood collected in EDTA vials and amniotic fluid collected in sterile vials were immediately processed for DNA extraction and B19V DNA detection by Real Time PCR (Artus Parvo B19 PCR Kit, Qiagen, Germany). In particular, 200 µL of whole blood and 400 µL of amniotic fluid, both added with 10 µL of an internal control, were extracted by QIAsymphony DSP DNA Mini Kit and QIAsymphony DSP Virus/Pathogen Midi Kit, respectively; 20 µL of DNA were used for B19 real‐time PCR, in accordance with the manufacturer's instructions. Quantitative real‐time PCR results were expressed in International Unit/mL (IU/mL).

Serum was collected in dedicated vacutainer serum separator tubes and immediately processed by automated chemiluminescence immunoassay (CLIA), on LIAISON Analyzer, to detect B19V IgG and IgM (LIAISON Biotrin Parvovirus B19 IgG Plus and LIAISON Biotrin Parvovirus B19 IgM, Diasorin SpA, Vercelli, Italy). An antibody index above or equal to 1.1 was considered positive.

Molecular and serological tests were performed following the manufacturer's instructions.

Electronic medical records were consulted for patients admitted to the intensive care unit (ICU) to investigate their clinical status and assess the presence of any comorbidities and past medical conditions. Congenital cardiac, hematological, metabolic, and malformation diseases were considered confounding factors. The diagnostic criteria for defining “severe disease” were those corresponding to the “major” and “catastrophic” levels described by Horn et al. [13].

A viral load > 4 log IU/mL and a compatible serological status (IgM−/IgG−; IgM+/IgG−; IgM+/IgG+) were considered to define a B19V primary infection.

Whole genome sequencing (WGS) and phylogenetic analysis were conducted on B19V DNA from 81 patients, resulting in positive findings from March–June 2024. To speed up the typing process while ensuring data reliability and accuracy, minimizing the risk of errors during analysis, a validated Parvovirus sequencing kit, Viral Surveillance Panel Kit (Illumina, San Diego, CA, USA), was used. The same DNA extracted and processed for real‐time PCR was used for WGS. The concentration and quality of DNA were measured and controlled with Qubit. Libraries were generated with Illumina index adapters and sequenced on a MiSeq instrument (Illumina, San Diego, CA, USA) with 2 × 150‐bp paired‐end reads. The reads obtained were analyzed using the DRAGEN software (Illumina, San Diego, CA, USA) and by an in‐house pipeline. Raw reads were trimmed for adapters and filtered for quality using Fastp (v0.23.2). Reference‐based assembly was performed with the BWA‐mem algorithm, aligning against the GenBank reference genome NC_000883.2. Single‐nucleotide polymorphism (SNP) variants were called with freebayes (v1.3.6), and all SNPS having a minimum supporting read frequency of 2% with a depth ≥ 10 were retained. To confirm the B19V genotype, a maximum likelihood (ML) phylogeny tree was performed with IqTree2 (v2.1.3) with 1000 replicates fast bootstrapping using the best‐fit model of nucleotide substitution TIM3 + F + R4.

Fisher's exact test was used to analyze categorical variables. Statistical analyses were carried out using SPSS 19.0 (SPSS Inc., Chicago, IL, USA) and a *p *< 0.05 was considered to be statistically significant. Fisher's exact test and *χ* ^2^ test for trend were used to estimate significant changes among different years.

## 3 Results

In our 600‐bed tertiary care pediatric hospital in Rome, from January 1, 2018, to December 31, 2024, 9695 blood samples were investigated for B19V DNA. The number of requests/samples for B19V DNA detection remained pretty stable at 100 to 130 requests per month until February 2024, with an increase from March to August 2024 and a peak of 221 requests in July.

**Figure 1:**
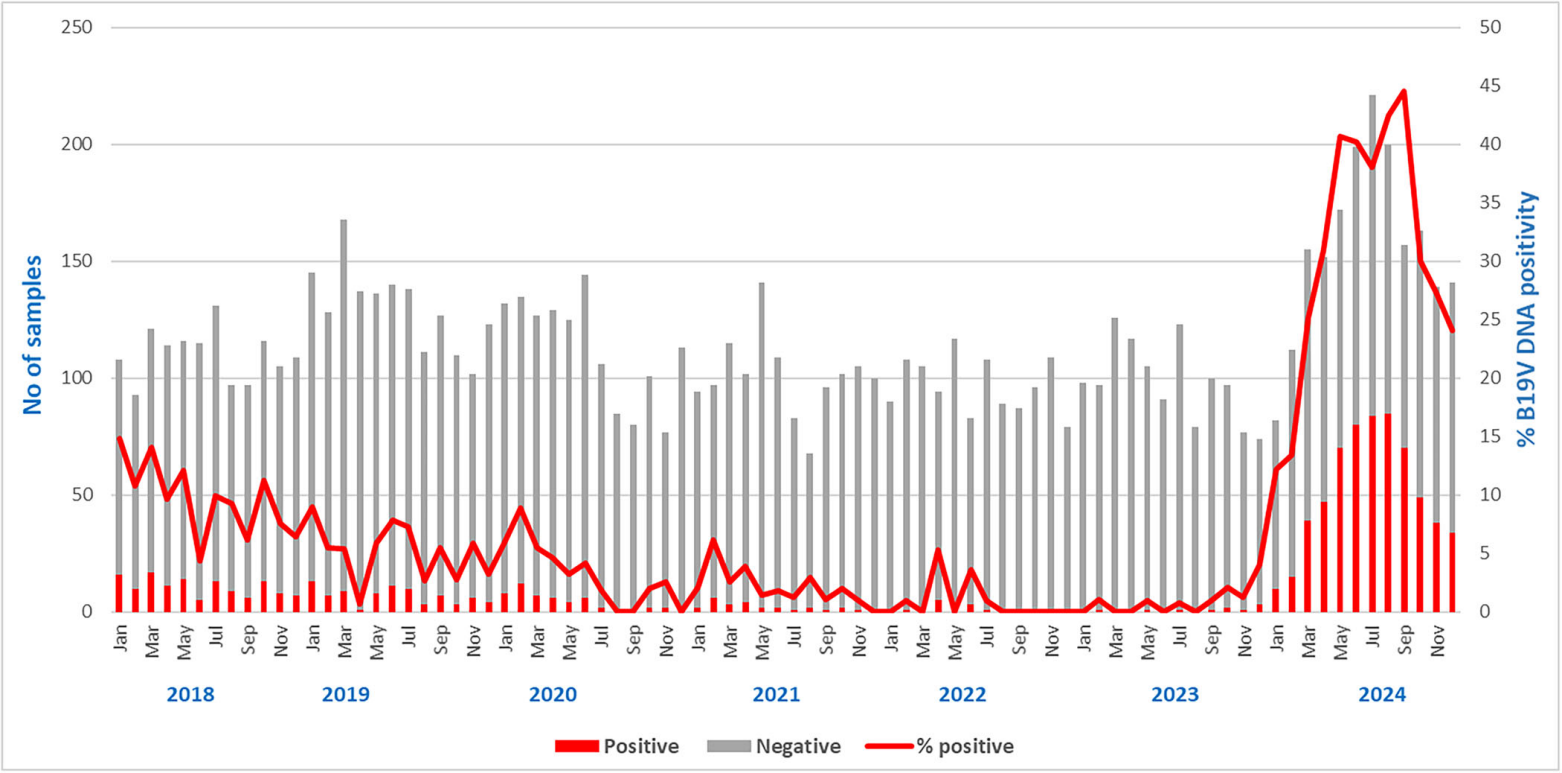
B19V DNA trend 2018*–*2024. The vertical axes indicate the number of samples analyzed and the percentage of positive samples detected over the period. The gray bar represents a negative result, and the red bar represents a positive one. The red line indicates the B19V DNA positivity rate.

Among the analyzed samples, 927 (9.6%) resulted positive. The annual positivity rate distribution over the years under study (Figure 1) was: 9.8% (129/1322) in 2018, 5.2% (82/1565) in 2019, 3.6% (49/1354) in 2020, 2.1% (26/1212) in 2021, 0.9% (10/1165) in 2022, 0.8% (10/1184) in 2023, and 32.8% (621/1893) in the year 2024 (*p* < 0.001).

**Figure 2:**
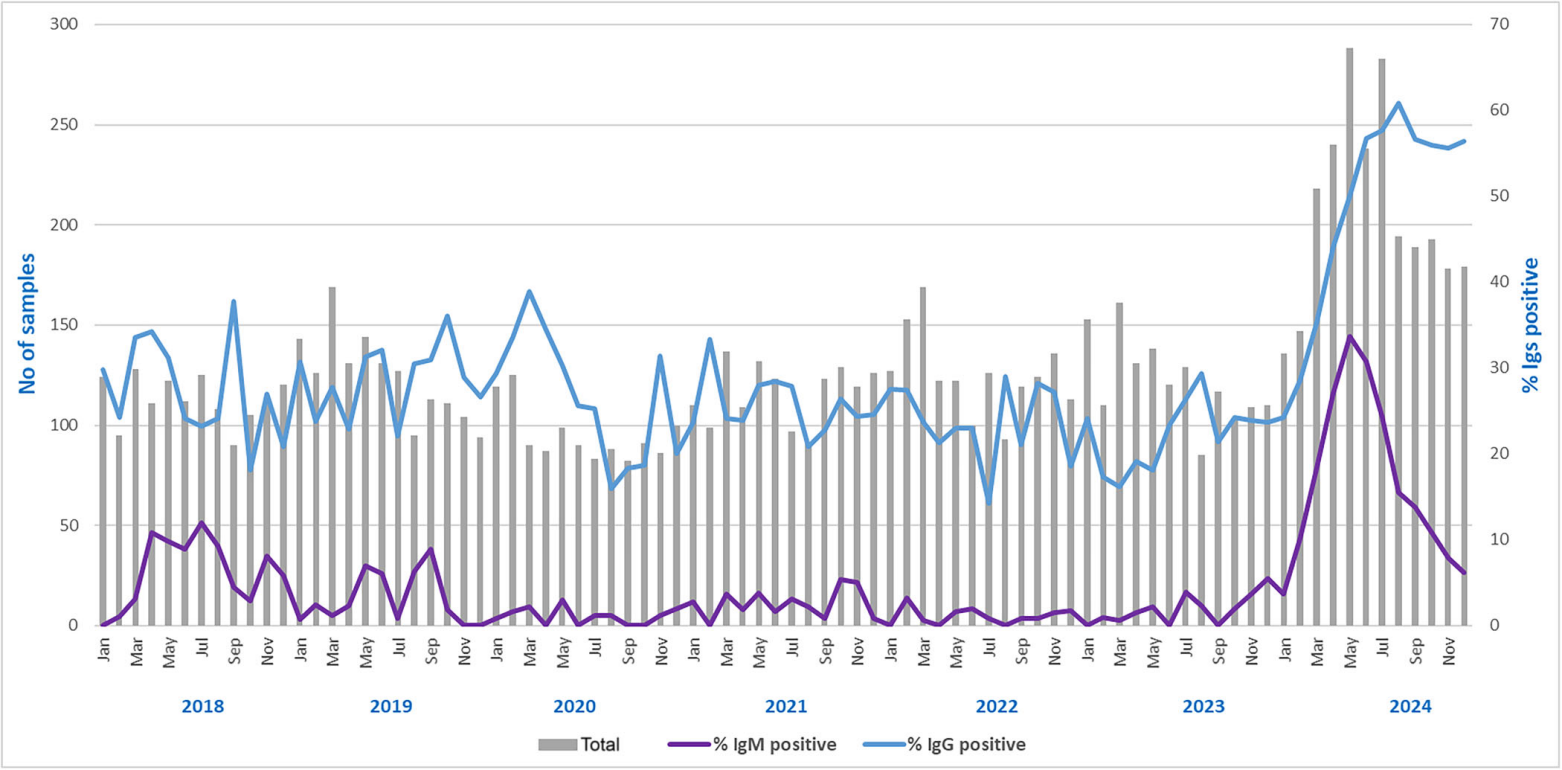
Anti‐B19V Igs trend 2018–2024. The vertical axes indicate the number of samples analyzed and the percentage of positive IgG (blue line) and IgM (violet line) samples detected throughout the period. The gray bar represents the number of samples investigated.

The same evaluation was carried out for anti‐B19V Immunoglobulins (Igs) detection on 10 827 serum samples. The positivity rate distribution of IgG and IgM show an increase in the last year (Figure 2): ranging from, respectively, 27.3% and 6.4% in 2018, 28.6% and 3.1% in 2019, 27.1% and 1.1% in 2020, 25.7% and 2.7% in 2021, 23.7% and 1.1% in 2022, 21.9% and 1.8% in 2023, and 49.7% and 18.8% in 2024 (*p* < 0.001).

Focusing on 2024, 621 blood samples referred to 314 patients (165 males and 149 females, median age 8.0 years, IQR 4.6–11.3) tested positive for B19V DNA.

**Figure 3:**
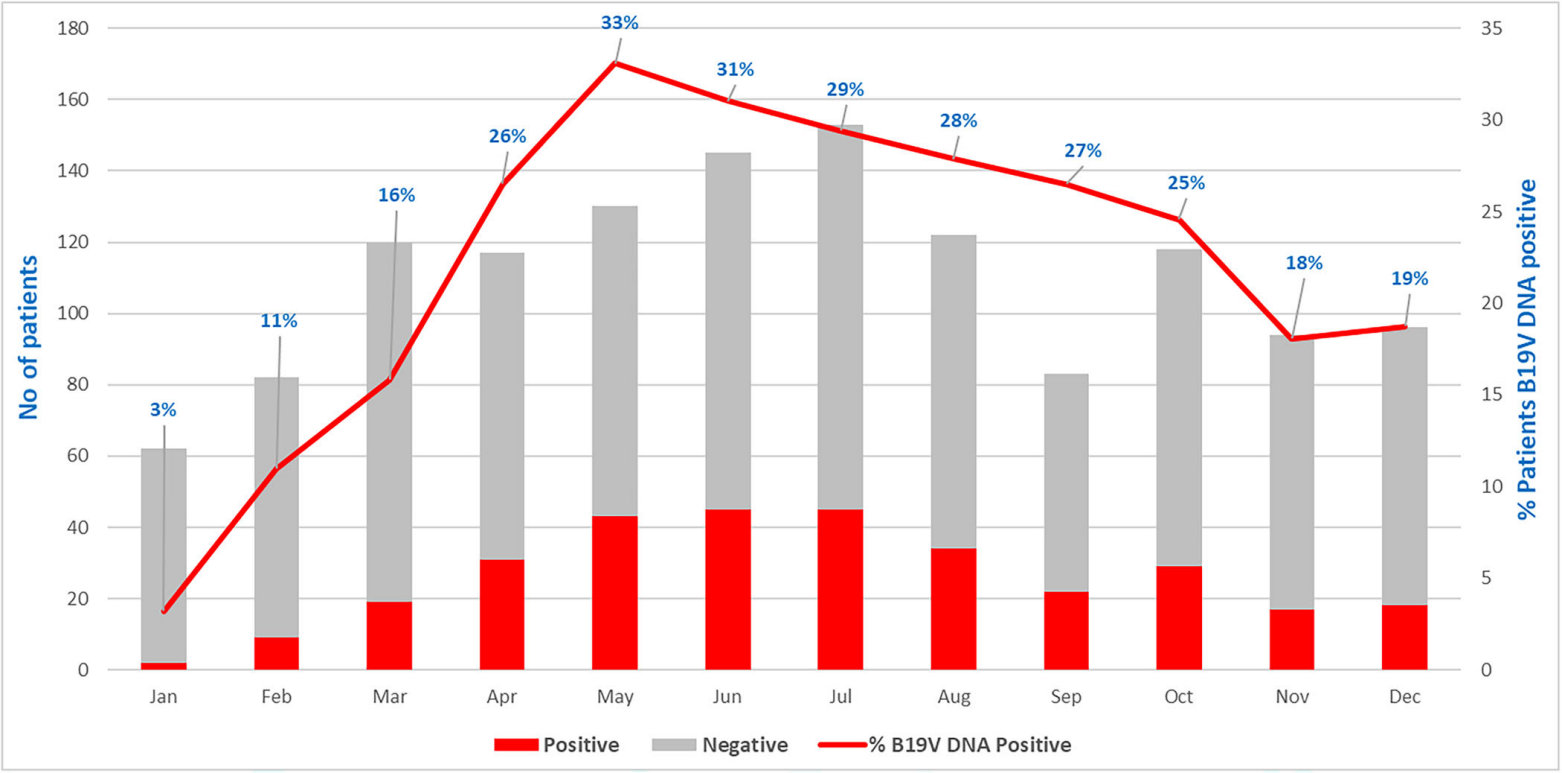
B19V DNA trend in 2024. The vertical axes indicate the number of patients investigated and the percentage of positive patients detected in 2024. The gray bar represents a negative result, and the red bar represents a positive one. The red line indicates the B19V DNA positivity rate.

Patients positive for B19V DNA detection remarkably increased over the months, from 3% in January to 33% in May (*p* < 0.001), to slowly decline in the following months (Figure 3).

In the same period, 2483 serum samples, referring to about 2000 patients, were also investigated for anti‐B19V IgM and IgG detection.

**Figure 4:**
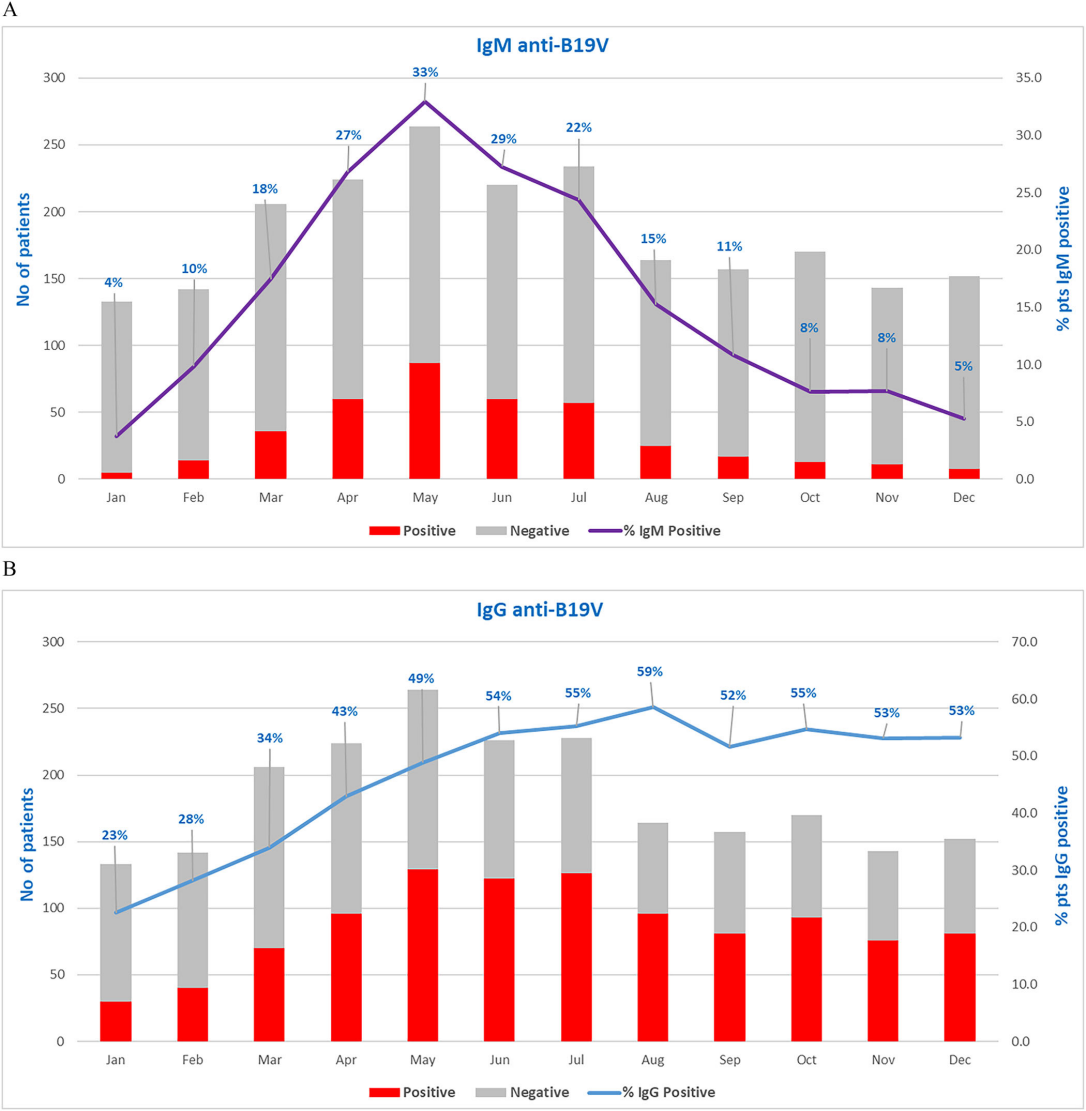
IgM (A) and IgG (B) anti‐B19V trend January–December 2024. The vertical axes indicate the number of patients investigated and the percentage of positive patients detected during the year 2024. The gray bar represents a negative result, and the red bar represents a positive one. Violet and blue lines indicate IgM and IgG anti‐B19V positivity rate, respectively.

Patients positive for IgM anti‐B19V were 393 (17.8%), 181 males and 212 females, with a median age of 7.7 years (IQR 4.6–11.1). The positivity for IgM sharply increased over the months, reaching a peak in May, rising from 4% to 33% (*p* < 0.001), and just as quickly decreased from then until December (Figure 4A).

Patients positive for IgG anti‐B19V were 1040 (41.9%), 503 males and 537 females, with a median age of 10.0 years (IQR 5.7–15.0). The positivity for IgG gradually increased over the months from 22.6% in January, reaching a stable level above 50% from May to December (Figure 4B).

Among the 314 patients resulted positive for B19V DNA detection in 2024, according to the defined criteria, 204 (65%) had a B19V primary infection (median age 7.7 years, IQR 4.4–11.1), 150/204 (73.5%) needed hospitalization, and 17/150 (11.3%) required ICU. In detail, 6/17 patients were males and 11/17 females (median age 3.4 years, IQR 1.2–7.4); 11/17 patients were admitted to the Emergency Department (ED) and 4/17 to the Cardiac ICU. For ICU patients, B19V DNA detection was required within 24 h from admission (14/17) and 2, 3, or 5 days later (3/17). From electronic medical records consultation, 8/17 patients presented clinical pre‐existing conditions. In particular, 3/8 patients presented blood disorders (two sickle cell disease and one spherocytosis): they showed very high viral loads (> 10^8^ IU/mL), were admitted to the ED for severe anemia and needed blood transfusion; their hospitalization lasted about 1 week. Two patients presented with congenital heart diseases and were admitted to the Cardiac ICU. One of them, heart transplanted, was admitted for suspected acute post‐infectious heart rejection; presented a viral load > 10^6^ IU/mL and a myocardial biopsy positive for B19V DNA (> 10^5^ IU/100 000 cells); his hospitalization lasted about 2 months. The other one was a 5‐month‐old child admitted for acute respiratory failure; she needed extracorporeal membrane oxygenation (ECMO), but her clinical conditions worsened and she died after 72 h of admission; B19V was the only pathogen detected (viral load > 10^5^ IU/mL). Two patients presented with congenital malformations. One, with a urinary tract malformation, was admitted for fever and acute gastroenteritis to the ED and then diagnosed with myocarditis; B19V was the only pathogen detected (viral load > 10^5^ IU/mL); his hospitalization lasted about 20 days. The other was a patient with craniofacial malformations, admitted to the ED for abdominal and pleural effusion and suspected meningitis; he was discharged after about 10 days with a diagnosis of sepsis due to B19V (viral load > 10^6^ IU/mL). The last patient with comorbidities was a Wilson's disease patient admitted for acute liver failure post II liver transplantation; she presented a B19V viral load > 10^8^ IU/mL, and was discharged after 6 months due to other complications.

Among patients admitted to the ICU, 9/17 presented no clinical pre‐existing condition. B19V viral load at first detection ranged from 10^4^ to 10^7^ IU/mL. Clinical outcome was: myocardial failure for 7/9 patients, 3/9 needed ECMO, and 1 of them died after 72 h of admission; 1 patient presented central nervous system involvement; 1 patient presented shock, ascites, and acute pancreatitis. Length of hospitalization was: 2–4 weeks (4 patients) and more than 1 month (4 patients).

Concerning amniotic fluid samples, 4/11 samples resulted positive. The first one was registered in November 2023 (viral load 10^7^ IU/mL) and three were registered in April, May, and August 2024 (viral load 10^2^ IU/mL, 10^7^ IU/mL, and 10^7^ IU/mL, respectively).

**Figure 5:**
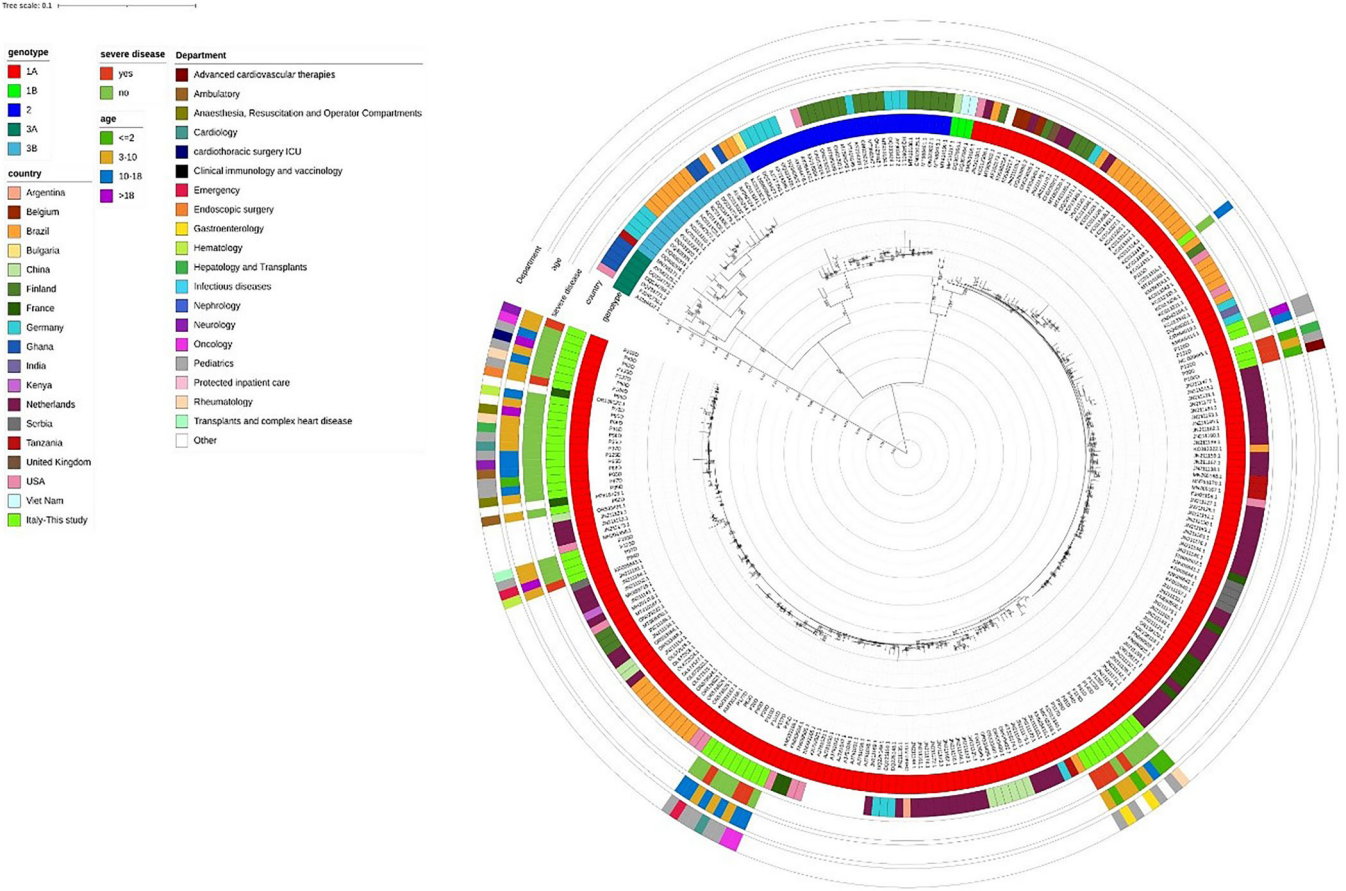
Estimated maximum likelihood phylogeny of the Parvovirus B19 sequences (*n* = 2328) and samples sequenced in this study (*n* = 50). Bootstrap values higher than 90 are shown on branches. Information regarding the samples is reported: genotype, country of isolation (in lime samples sequenced in this study), disease severity, age, and department.

The WGS of Parvovirus B19 was performed on DNA from 81/314 patients and was positive. Of these, 31 were excluded from phylogenetic analysis due to a low number of reads. The viral genome analysis of the remaining 50 samples, including 11/17 from patients admitted to ICUs, showed that only subtype 1A was circulating in our study population. The distribution of Parvovirus B19 sequences against clinical/demographic characteristics and against the global context of B19 (reference sequences deposited on NCBI [National Center for Biotechnology Information], Bethesda (MD): National Library of Medicine (US), National Center for Biotechnology Information; [1988]. Available from: https://www.ncbi.nlm.nih.gov/] is shown by the ML Tree in Figure 5. A low genetic variability was observed between the strains from severe and mild disease, suggesting a limited intragenotypic diversity of Parvovirus B19 in this study. This finding is consistent with the presence of only subtype 1A. In fact, when analyzing SNPs responsible for nonsynonymous mutations among samples with severe disease versus mild disease, two mutations (L57F and C17S) in the nuclease domain of nonstructural protein (NS1) were found to be more frequent in strains from patients with severe disease compared to strains from mild disease (*p* = 0.036 and 0.038, respectively). No significant differences in demographic and clinical characteristics were observed.

## 4 Discussion and Conclusions

As previously described and declared in a “threat assessment brief” from ECDC, nine EU/EEA countries have reported increased detections of B19V on the European surveillance portal for infectious diseases, EpiPulse, from a number of monitoring systems, mostly during late 2023 and early 2024 [14, 15, 16]. Italy was not among the Member States that reported information through EpiPulse, but the results of this study confirm this trend.

Indeed, first reports regarding B19V circulation describe an initial surge in France and Israel in the spring of 2023 [17, 18], not detected in our population. At Bambino Gesù Children's Hospital, a significant increase in the circulation of B19V was observed starting from January 2024, both in comparison with the period of low incidence, corresponding to the COVID‐19 pandemic, and in comparison with the prepandemic period.

In this study, serological, molecular, and typing data were used to provide an overview of the circulation of B19V in a representative number of patients (more than 90% pediatric), in order to characterize the epidemiology and clinical impact of this rebound, when the infection revealed particularly severe.

Throughout 2018–2024, in the study setting, the number of requests for B19V DNA detection remained constant until February 2024, ranging from 100 to 130 requests/month. It increased in the following months, reaching a peak of 221 requests in July, due to greater awareness by clinicians and patients' follow‐up. However, the annual positivity rate was not evenly distributed over the years evaluated: 9.8% in the previous epidemic period (2018), 0.8% in the SARS‐CoV‐2 pandemic period (2023), and 32.8% in the current outbreak (2024). Although B19V incidence peaks every few years, the current surge is the most relevant to date: in May 2024, B19V DNA assays resulted positive in 41% of tested samples versus the last previous peak of about 15% registered in January 2018 (Figure 1). The same trend resulted from the analysis of the IgM anti‐B19V positivity rate, which was always under 5% in the previous years, except for 2018 (6.4%), with a sharp increase in 2024 from 4% in January to 33% in May and an equally rapid reduction to 5% in December. This Parvovirus B19 rebound reflected in a change of IgG anti‐B19V seroprevalence, with a reduction in the positivity rate from 27.3% in 2018 to 21.9% in 2023, due to low virus circulation, particularly during the COVID pandemic, and a marked and steady increase to above 50% from May 2024 onward.

Notably, only the analysis of IgM trend allows for unambiguously tracing the 2024 peak in B19V circulation in May (Figures 2 and 4A). Analyzing B19V DNA trend, to represent a real picture of the epidemiology, it is necessary to evaluate patients, in which the peak in May can be observed (Figure 3). When analyzing requests, a bias is evident, as the persistence of the B19V DNA viral load, detected in patients' follow‐up, maintains the trend of positivity above 40% until September, with a decrease in the last quarter of the year (Figure 1). The analysis of the IgG trend highlights overcoming the immune gap.

Considering the broad spectrum of clinical manifestations due to B19V infection, intentionally, no exclusion criteria were applied to the study population. Actually, results show that B19V circulation has consequences both in fragile hospitalized patients as well as in the general population, where virus diffusion is larger than registered, but underestimated by the lack of a surveillance system in Italy and in other European countries.

The current B19V outbreak revealed quite a serious issue: unexpectedly, among patients with primary infection, 73.5% (150/204) were hospitalized, 11.3% of whom (17/150) needed ICU. In these most severe patients, clinical outcomes were myocardial failure (10/17), with two deaths, severe anemia (3/17), central nervous system disease (2/17), liver failure (1/17) and shock, ascites, and acute pancreatitis (1/17). Coherent with the known effects of B19V primary infection, all patients with severe anemia presented congenital blood disorders.

This rebound of B19V infection was associated with particularly serious multiorgan diseases, not always related to previous pathologies. Indeed, nine patients admitted to the ICU did not have any pre‐existing clinical condition or underlying disease, including one of the two patients who died of fulminant myocarditis. Concerning the nine patients with myocarditis, four of them underwent myocardial biopsy, resulting in positive B19V DNA detection. Acute myocarditis is a life‐threatening condition in children, with viral infections being the most common cause [19]. Poeta and colleagues describe 65 cases of pediatric myocarditis in Italy in 2024, half of which were in children with B19V infection, identified through noninvasive methods (serology and DNA blood testing). Also in their cohort, a high number of patients were immunocompetent [20, 21]. In this study, comorbidities evaluation was carried out only for the 17 patients with the most severe clinical outcome, and although it is likely that concomitant diseases may worsen B19V infection, this correlation does not seem so evident (8/17 patients with comorbidities and 9/17 without).

The high B19V circulation and the rise of awareness among clinicians could explain the current relative increase in infection diagnosis in pregnant women. In the last years (2023–2024), the Bambino Gesù Fetal and Perinatal Medicine and Surgery Center evaluated 11 amniotic fluid samples for suspected B19V infection, four of which resulted positive. The first was registered in November 2023, and three were registered in April, May, and August 2024. Fetal echography revealed a fetus with cardiomyopathy in the first patient, a fetus with lower biometrics in the second, and a fetus with transposition of large vessels and lower biometrics in the third patient; unfortunately, no information was available for the fourth fetus. A marked increase in B19V infection in pregnant women in 2024 has also been reported by an obstetrics referral center in Northern Italy [22].

As is known, B19V is classified into three distinct genotypes, 1, 2, and 3, differing from each other by 2%–3%. Predominance of genotype 1 across all the continents is seen; in Europe, genotype 1 represents 61.4%, followed by genotype 2 (36.73%), and quite rarely, genotype 3 (1.62%) [2]. There is only a single serotype that covers all three genotypes, as suggested by antibody cross‐reactivity [23]. In the study population, typing of 81 B19V DNA detected in 2024 revealed exclusively subtype 1A; further analysis is needed to shed light also on potential evolutionary trends, responsible for the current epidemiological dynamics.

Although to date, no particular associations between different genotypes and clinical conditions have been described in B19V infection [2], the large number of positive patients and the morbidity that characterize the current rebound, make worthwhile the genetic characterization of the parvovirus strains currently circulating to evaluate possible correlations with the most severe clinical manifestations. The phylogenetic analysis conducted in this study showed no significant differences in demographic and clinical characteristics, confirming that, also in this 2024 rebound, there was no correlation between Parvovirus B19 genotype and disease severity. However, to support the hypothesis that subtle intra‐genotypic differences (such as SNPs), rather than large genomic differences, might play a role in disease pathogenesis, in‐depth characterization studies are required. As reported in the results, a couple of polymorphisms in the nuclease region of NS1 of the Parvovirus genome are associated with a more severe disease. This finding needs to be supported by more extensive studies, including the evaluation of viral polymorphisms in strains circulated in previous years, and those that will circulate in the near future, to highlight potential implications for clinical outcome, as well as for reinfection risk and immune escape.

A limitation of this study is that it is a single‐center assessment, representing a local picture of B19V circulation, which is coherent with what has recently been described in national surveillance reports from several European countries [15].

However, it provides an overall view of B19V infection in a large pediatric population, from 2018 to the present, describing its epidemiology and more challenging clinical involvement.

In conclusion, during such a remarkable epidemic outbreak, the virus should be considered as a possible cause of disease even when the clinical picture is not typical of B19V infection, especially in fragile patients and in all conditions in which there are symptoms (at the cardiac level, but not only) that cannot be related to other pathogens. Moreover, since B19V infection can also be asymptomatic or paucisymptomatic, in periods of such high incidence, the screening of healthcare workers caring for vulnerable subjects could probably be considered to prevent any nosocomial transmission and fatal consequences. In this regard, our data support the use of serological tests, which are cheaper, quicker, and easier to perform than molecular assays, as a valuable tool to promptly intercept possible outbreaks of infection among family members and caregivers.

## Author Contributions

**Stefania Ranno:** conceptualization of the study, analysis and interpretation of data, writing and editing of the manuscript. **Luana Coltella:** conceptualization of the study, analysis and interpretation of data, writing and editing of the manuscript. **Luna Colagrossi:** typing and data analysis, support writing. **Valeria Fox:** typing and data analysis, support writing. **Cristina Russo:** review and editing. **Alberto Villani:** review and editing. **Leonardo Caforio:** review and editing. **Carlo Federico Perno:** review and editing. All authors read and approved the final version of the manuscript.

## Ethics Statement

This is a retrospective observational study in which results are collected anonymously and in aggregate form. The 17 patients focused on are reported in pseudonymous form, and no direct or indirect identification is possible. The study has been formally notified to the Ethics Committee and approved (No. 3537/2024).

## Consent

The authors have nothing to report.

## Conflicts of Interest

The authors declare no conflicts of interest.

## Data Availability

The data sets used and/or analyzed during the current study are available from the corresponding author on request.
